# Childhood temperament predictors of adolescent physical activity

**DOI:** 10.1186/s12889-016-3998-5

**Published:** 2017-01-05

**Authors:** James A Janssen, Jacek Kolacz, Lilly Shanahan, Meghan J. Gangel, Susan D. Calkins, Susan P. Keane, Laurie Wideman

**Affiliations:** 1 Department of Kinesiology, University of North Carolina Greensboro, Greensboro, NC 27402 USA; 2 Department of Psychology and Neuroscience, University of North Carolina Chapel Hill, Chapel Hill, NC 27599-3270 USA; 3 Department of Psychology & Jacobs Center for Productive Youth Development, University of Zurich, CH - 8050 Zurich, Switzerland; 4 Department of Psychology, University of North Carolina Greensboro, Greensboro, NC 27402 USA; 5 Department of Human Development and Family Studies, Greensboro, NC 27402 USA

**Keywords:** Temperament, Physical activity, Childhood, Adolescence, Longitudinal

## Abstract

**Background:**

Physical inactivity is a leading cause of mortality worldwide. Many patterns of physical activity involvement are established early in life. To date, the role of easily identifiable early-life individual predictors of PA, such as childhood temperament, remains relatively unexplored. Here, we tested whether childhood temperamental activity level, high intensity pleasure, low intensity pleasure, and surgency predicted engagement in physical activity (PA) patterns 11 years later in adolescence.

**Methods:**

Data came from a longitudinal community study (*N* = 206 participants, 53% females, 70% Caucasian). Parents reported their children’s temperamental characteristics using the Child Behavior Questionnaire (CBQ) when children were 4 & 5 years old. Approximately 11 years later, adolescents completed self-reports of PA using the Godin Leisure Time Exercise Questionnaire and the Youth Risk Behavior Survey. Ordered logistic regression, ordinary least squares linear regression, and Zero-inflated Poisson regression models were used to predict adolescent PA from childhood temperament. Race, socioeconomic status, and adolescent body mass index were used as covariates.

**Results:**

Males with greater childhood temperamental activity level engaged in greater adolescent PA volume (B = .42, SE = .13) and a 1 SD difference in childhood temperamental activity level predicted 29.7% more strenuous adolescent PA per week. Males’ high intensity pleasure predicted higher adolescent PA volume (B = .28, SE = .12). Males’ surgency positively predicted more frequent PA activity (B = .47, SE = .23, OR = 1.61, 95% CI: 1.02, 2.54) and PA volume (B = .31, SE = .12). No predictions from females’ childhood temperament to later PA engagement were identified.

**Conclusions:**

Childhood temperament may influence the formation of later PA habits, particularly in males. Boys with high temperamental activity level, high intensity pleasure, and surgency may directly seek out pastimes that involve PA. Indirectly, temperament may also influence caregivers’ perceptions of optimal activity choices for children. Understanding how temperament influences the development of PA patterns has the potential to inform efforts aimed at promoting long-term PA engagement and physical health.

## Background

The prevalence of chronic diseases linked to physical inactivity and sedentary behaviors—such as cardiovascular disease and diabetes—continues to rise. And, these diseases have their onsets at increasingly younger ages [1–5]. Unhealthy lifestyle behaviors, including physical inactivity, sedentary behavior, and poor nutrition have been implicated as key players in the development of chronic disease [4, 5]. Physical inactivity in particular is the fourth-leading cause of mortality worldwide [6]. Characteristic patterns of physical activity (PA) are typically established during the early life course and remain relatively stable over time [7–10]. Therefore, the identification of predictors of PA engagement during the early lifespan is crucial, and could aid in the development of successful strategies aimed at increasing PA levels, and, ultimately, minimizing chronic disease risk.

Previous research has established that age, race, socioeconomic status, and a range of sociocultural factors predict youth engagement in PA [11–16]. Theoretical frameworks in the developmental sciences and child development literature also support the idea that children’s individual characteristics that are primarily based within the person—such as their temperament—shape their developmental contexts in important ways, including the activities that they engage in [e.g., 17, 18]. However, childhood temperament remains relatively unexplored as a predictor of PA.

Yet, early within-person factors such as temperament likely play an important role in long-term patterns of PA engagement given that patterns of physical activity are often carried forward into adulthood [8–10]—despite frequent changes in social contexts (e.g., changes in friends) and physical environments (e.g., moving to different schools or places). Here we examine long-term predictions from childhood temperament to adolescent PA in an effort to better understand childhood temperament as one potential source of continuity, or “tracking,” of PA despite the many changing social and physical contexts that children encounter during the early lifespan.

Temperament refers to children’s inter-individual differences in physiological, behavioral, and emotional reactivity and self-regulation that are reflected in the speed/intensity of responses to stimuli, and recovery from such responses. These individual differences are relatively stable throughout development and are typically expressed across contexts [19]. Temperamental differences can be observed beginning early in life, likely have biological bases, and create a foundation for shaping personality and behavior into adolescence and adulthood [20]. Temperament reflects generalized tendencies [21] and is therefore distinct from situational motivation (e.g., exercising to build muscle or lose weight) and abilities (e.g., hand-eye coordination that is needed in many sports).

Temperament is conceptualized along a variety of dimensions. Here we focus on four dimensions of temperament with potential to influence PA in youth. A summary of these dimensions and example items to measure them can be found in Table 1. First, temperamental activity level (TAL) refers to a child’s gross level of activity and includes both rate and extent of locomotion during activities of daily living (e.g., a tendency to run versus to walk). Second, high intensity pleasure (HIP) is a dimension that refers to the amount of pleasure or enjoyment that children experience in situations involving high stimulus intensity, rate, complexity, novelty, and incongruity. High intensity pleasure is associated with children’s differences in optimal levels of arousal [20, 22], and, thus, drives youth to seek out activities that satisfy their preferred levels of arousal. Third, low intensity pleasure (LIP) refers to the amount of pleasure or enjoyment that children experience in situations involving low stimulus intensity, rate, complexity, novelty, and incongruity. For example, children who score high on LIP may enjoy gentle swinging on the playground. HIP and LIP are distinct dimensions of temperament: Children may be drawn to either high- or low-intensity activities or they may enjoy both. Accordingly, measures of HIP and LIP tend to have low or no correlations [23]. Finally, surgencycm (SUR) is a higher-order temperamental trait that subsumes several individual dimensions of temperament: activity level, impulsivity, shyness, and high intensity pleasure. Children high in surgency are highly active, impulsive, and quick to respond. In contrast, children low in surgency are low in activity, impulsivity, and HIP, and higher in shyness [20]. Surgency’s integration of multiple individual dimensions of temperament may provide insight into childhood temperament above and beyond more circumscribed individual dimensions.

**Table 1:** Temperament dimensions, definitions, and example items from the Child Behavior Questionnaire (CBQ)

Dimension	Definition	Example items
Temperamental activity level (TAL)	Gross motor activity including the rate and extent of the locomotion	Tends to run rather than walk from room to room
		Is full of energy, even in the evening
High Intensity Pleasure (HIP)	Amount of pleasure related to situations involving high stimulus intensity, rate, complexity, novelty, and incongruity	Likes going down high slides or other adventurous activities
		Enjoys activities such as being chased, spun around by the arms, etc.
Low Intensity Pleasure (LIP)	Amount of pleasure involved in situations with low stimulus intensity, rate, complexity, novelty, and incongruity	Enjoys taking warm baths
		Enjoys just being talked to
Surgency (SUR)	A trait aspect of emotional reactivity marked by rapid approach to rewards and high activity level	Computed from measures of Impulsivity, High Intensity Pleasure, Activity Level, and Shyness (negative loading)

To our knowledge, only one study has previously investigated associations between temperament and PA [24]. This study of females aged 8–12 years old showed that higher levels of temperamental activity were associated with higher levels of energy expenditure (assessed by doubly labeled water), and lower levels of body fat. This suggests that temperamental activity level is associated with individual differences in PA. However, the study was cross-sectional in design, and, therefore, uninformative about predictions from temperament to PA engagement over time. In addition, the study was limited to enrollment of females of normal weight only and did not include a direct measure of PA engagement.

However, links between temperament and direct measures of PA may be multifaceted. Indeed, given that temperament is a reflection of the speed and intensity of behavioral responses to stimuli, it is possible that links between temperament and PA may differ depending on the intensity of PA. For example, slow rhythmic PA movements may not elicit the preferred arousal level of a child with high surgency, or a child with a propensity for high intensity pleasure. Accordingly, studies of the associations between temperament and PA should include measures of PA that represent a range of intensities of PA.

Addressing gaps in extant research, the main goal of the current study was to test associations among multiple dimensions of childhood temperament and adolescent self-reported PA. In doing so, we used an existing prospective-longitudinal study of males and females from childhood to late adolescence, with measures spanning over one decade. We used two measures of self-reported PA (one ‘usual’, one ‘past week’) to allow for interpretation of total PA volume, number of days of PA per week, and number of strenuous bouts per week. We hypothesized that: a) childhood temperamental activity level, high intensity pleasure, and surgency would positively predict the total volume of adolescent PA; b) childhood low intensity pleasure would negatively predict adolescent PA; and c) childhood high intensity pleasure would predict higher levels of adolescent strenuous PA. In addition, we aimed to examine whether the associations between childhood temperament and adolescent PA differed by sex. The novelty of this exploratory research question limited our ability to establish sex- specific hypotheses.

## Methods

### Participants

Participants were drawn from the RIGHT (Research Investigating Growth and Health Trajectories) Track study, an ongoing prospective-longitudinal community study. Children and their caregivers were recruited at age 2 through child day care centers, the County Health Department, and the local Women, Infants, and Children program. Recruitment targeted a sample that was racially and socioeconomically representative of the surrounding community.

The current study examined a subset of *N* = 206 participants with data on childhood temperament and adolescent PA (females = 53%; Caucasian = 70%). Socioeconomic status was assessed using the Hollingshead Index, a weighted average of parental education and employment [25]. Scores ranging from 40 to 54 represent middle class professional and technical occupations. Families from a range of socio-economic strata typically captured by this scale are represented in this analysis (Hollingshead score M = 41.89, SD = 9.79, Min = 14, Max = 16). For a detailed description of the study from which this sample was drawn, please see [26].

### Procedures

At ages 4 and 5 years (2001–2004), families visited the university laboratory; average age was 4.38 years (SD = .27) at the first and 5.62 years (SD = .24) at the second visit. During these visits, parents (typically mothers) completed survey measures assessing their child’s temperament and their family SES. At 15 and 16 years (2012–2015), participants returned to the university laboratory and completed self-reported PA assessments. During this visit, trained interviewers measured height and weight.

### Measures

Adolescent self-reported physical activity. Physical activity measures were used to assess frequency and reported intensity of adolescent PA. The Youth Risk Behavior Survey (YRBS) [27] provided a measure of the number of days in which PA approximates daily PA guidelines. A single PA item with 8 response options was taken from the YRBS survey: ‘During the past 7 days, on how many days were you physically active for a total of at least 60 min per day’? Based on the number of days, participants were classified as inactive (0 days per week), somewhat active (0–4 days per week), or very active (5–7 days per week).

The Godin Leisure-Time Exercise Questionnaire [28, 29] was used to measure PA according to intensity and also to provide a global assessment of PA volume. The Godin Leisure-Time Exercise Questionnaire has been shown to have acceptable validity and reliability in a range of settings [29]. Participants reported the number of times per week, on average, during which they engaged in strenuous, moderate, and mild PA for more than 15 min. Strenuous physical activity was defined to participants as activity in which the heart beats rapidly. Examples provided to participants included running and football. Moderate physical activity was defined as non-exhausting activities; examples included baseball and fast walking. Mild physical activity was defined as that requiring minimal effort, such as yoga or archery.

To avoid biased parameter estimates caused by outlying cases, strenuous and mild PA variables were transformed by winsorizing extreme values above 7 (mild *n* = 4, strenuous *n* = 4). A total Godin score was calculated by weighting each type of PA using the equation [9*Strenuous] + [5*Moderate] + [3*Mild] [28]. The resulting values were adjusted for skewness via a natural log transformation and standardized for ease of interpretation. Both the Godin Leisure-Time Exercise Questionnaire and the YRBS have been shown to have acceptable reliability and validity for PA assessment in youth [29, 30].

Childhood temperament was assessed using maternal reports on the Children’s Behavior Questionnaire Long Form (CBQ-LF) [31]. This validated 195-item questionnaire assesses children’s typical behavior within the prior 6 months on a 7-point Likert-type scale ranging from 1 (“Extremely untrue of your child”) to 7 (“Extremely true of your child”) [31]. Questions are designed to aggregate children’s behavior across a variety of contexts and stimuli/events. The subscales used for this study were 1) temperamental activity level (TAL); 2) high intensity pleasure (HIP); 3) low intensity pleasure (LIP); and 4) surgency (SUR). Cronbach’s alpha for these subscales showed good internal consistency (Average age 4 & 5 TAL α = .77, HIP α = .79, LIP α = .72) and were comparable to previously reported age 4 & 5 values (TAL α = .75, HIP α = .79, LIP α = .64) [31]. Each of these dimension of temperament was highly stable between ages 4 and 5 (TAL (Age 4, 5) *r* = .79, HIP (Age 4, 5) *r* = .71, LIP (Age 4, 5) *r* = .66, SU (Age 4, 5) *r* = .82) and thus were averaged across these two time points.

Covariates. Body mass index (BMI) was computed using the formula weight/(height^2^). Height (kg) and weight (m) at age 15/16 were measured by trained interviewers during participants’ visits to the laboratory. Height was measured to the nearest 0.1 cm with a wall mounted, calibrated stadiometer (SECA, Chino CA). Weight was measured to the nearest 0.1 kg with a balance-beam scale (Detecto-medic, Brooklyn NY). Self-reported height and weight were used for participants who wished to participate, but were unable to attend the laboratory for the adolescent visit. For participants who completed both laboratory visits and self-reported their BMI, the two measures were highly correlated (*r* = .92, *p* < .001). Furthermore, we did not observe significant differences between laboratory-based and self-report-based BMI calculations (paired samples *t*-test; t(157) = −1.621, *p*−.11), suggesting that BMI calculations based on self-reports were not biased toward under- or overestimation. The Hollingshead Index [25] was used to assess the socioeconomic status (SES) of the participating families at ages 4 and 5 (*r* = .80, *p* < .001 for the age 4, 5 SES correlation). Hollingshead scores were derived by a weighted average of parental education and employment [25] and can range from 8 to 66. Parents reported children’s race/ethnicity at the onset of the study.

## Analytic strategy

All statistical modeling was conducted using SAS 9.4 (SAS Institute, Cary NC). First, analyses predicting categories of PA involvement per week were estimated using ordered logistic regression models (also known as ordinal logistic regressions). Parameters resulting from these models were converted to odds ratios using an e^x^ transformation. The resulting odds ratios represent the change in the odds of being in the next higher frequency PA group that corresponds to a 1 SD increase in the continuous predictor. The appropriateness of these models was assessed using a Proportional Odds Assumption Chi-Square test, wherein a non-significant (*p* > .05) value suggests that the ordered logistic regression is acceptable for describing the associations between the predictors and dependent variable responses [32].

Second, models predicting the continuous total Godin PA score were estimated using ordinary least squares linear regression models. Explanatory power for these models was assessed by the amount of variance explained in the dependent variable (R^2^). Third, models predicting strenuous PA with child temperament used Zero-Inflated Poisson regressions. These models were selected in order to best reflect the count distribution of this variable with a high frequency of zero responses. The adequacy of these models in explaining the dependent variable was assessed with a Chi-Square test. A non-significant value (*p* > .05) suggests that the observed and expected dependent variable values are not significantly different from each other and, thus, the model fits the data well. Poisson regression parameters can be converted to change scores that represent the percent change in the number of bouts of strenuous PA that result from a one SD increase in the continuous predictor.

In all models, covariates (race, SES, and adolescent BMI) were entered first. Next, separate models were estimated for each temperament dimension. All models were estimated for males only, females only, and for the full sample using interaction terms to examine whether temperament-PA associations differed by sex. Interactions were represented by including the main effects of the respective temperament dimension and sex, and a temperament dimension X sex interaction term.

Missing data. Of the participants who were seen at age 2, data on childhood temperament data was available for 91% of children. Due to attrition over the course of 11 years, adolescent physical activity variables were available for a smaller subset of participants. Over 80% of the original sample participants, who were in the study at ages 4 and 5, had at least one adolescent PA assessments over a decade later at age 16. Seventy-one percent of the sample had data on days of physical activity, 63% on Strenuous PA, and 58% on the Godin score (Godin score could not be calculated if mild or moderate PA self-reports were missing).

Attrition analyses revealed that participation in adolescence was unrelated to race (*p* = .42), and marginally related to male sex (*p* = .05) and higher SES (*p* = .05), a finding that is not uncommon in long-term longitudinal studies focused on health [e.g., 33]. Sex and SES could be considered missing at random and ignorable provided that they are included as covariates in our models [34]. The results reported below are based on models estimated using ordinary least squares, which uses listwise deletion in cases with missing data. Note, however, that the reported results did not substantively differ when the data were re-analyzed using a full information maximum likelihood estimator, which uses all available data values [35]. Thus, we did not find evidence that the listwise deletion method for missing values biased the model results.

## Results

**Fig. 1:**
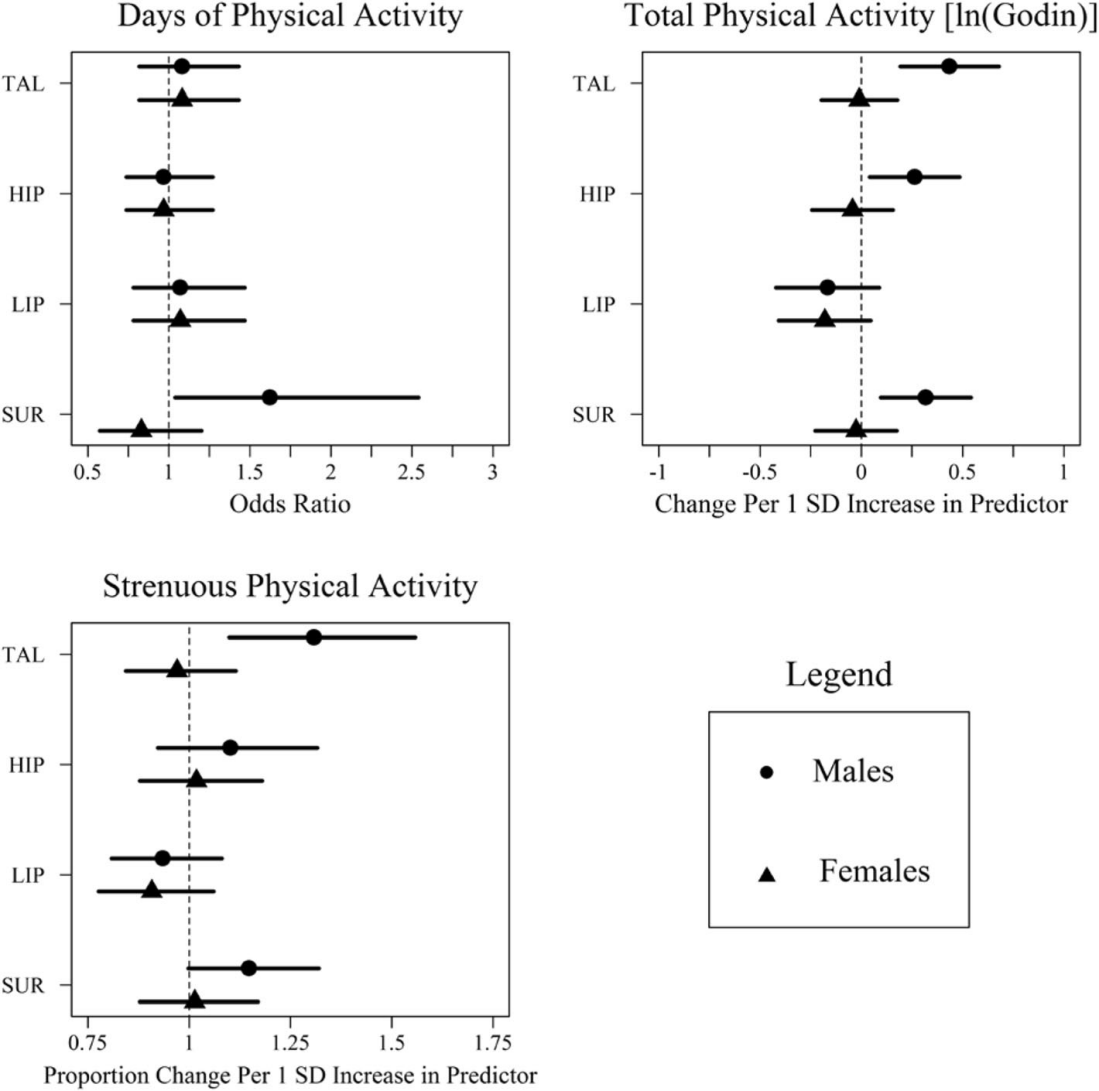
Model results for temperament dimension predicting physical activity outcomes; horizontal lines represent 95% confidence intervals; when a horizontal line touched the dashed vertical line, the prediction is non-significant (*p* > .05)

Descriptive statistics for all continuous variables in our analytic sample are presented in Table 2. Average levels of temperamental activity level, high intensity pleasure, and surgency were higher in males than in females. Conversely, average levels of low intensity pleasure were higher in females than in males. Males engaged in more bouts of strenuous PA than females; however there were no sex differences in overall PA volume (Godin Score). Based on the YRBS item, 17% of participants reported 0 days of PA, 35% reported 1–4 days, and 48% reported 5 or more days. There was a trend toward more days of weekly PA engagement in males than in females (Wilcoxon-Mann—Whitney *U* = 9621.50, *p* = .068). Table 3 shows bivariate correlations among all study variables. All measures of PA were moderately to strongly correlated with one another in both males and females. Temperamental activity level, high intensity pleasure, and surgency were moderately to strongly correlated in both males and females. Low intensity pleasure was negatively correlated with temperamental activity in males but was not significantly associated with any other dimensions of temperament in either sex. Results from all models testing associations between childhood temperament and adolescent PA engagement are presented numerically in Table 4 and graphically in Fig. 1.

**Table 2:** Descriptive statistics of childhood temperament and adolescent physical activity variables

	Overall (*N* = 206)		Males (*N* = 89)		Females (*N* = 117)		Sex Difference^a^
Variable	*M*	*SD*	*M*	*SD*	*M*	*SD*	
*Childhood (4/5 years)*							
SES	41.89	9.79	42.68	9.10	41.21	10.34	
TAL	4.96	0.70	5.15	0.64	4.80	0.72	**
HIP	5.11	0.74	5.31	0.73	4.95	0.72	**
LIP	5.49	0.58	5.27	0.57	5.67	0.52	**
SUR	4.87	0.64	5.01	0.66	4.74	0.60	**
*Adolescence (15/16 years)*							
Strenuous PA^b^	3.22	3.05	4.11	3.67	2.53	2.29	**
Godin Score	51.84	38.91	61.01	47.75	44.91	29.17	
BMI	24.15	6.20	24.02	6.42	24.24	6.06	
Days of PA^c^	**%**		**%**		**%**		
0 days	16.9		12.5		20.0		^†^
1-4 days	35.0		31.8		36.0		
5-7 days	48.1		55.7		44.0		

^†^ *p* < .10, *p < .05, **p < .01; PA = Physical activity; SES = Socioeconomic status; TAL = Temperamental activity level; HIP = High intensity pleasure; LIP = Low intensity pleasure; SUR = Surgency; ^a^ independent-samples *t*-test, except where noted; ^b^results of *t*-test did not substantively differ whether variable was raw or winsorized; ^c^Wilcoxon-Mann—Whitney *U* test statistic

**Table 3:** Bivariate Pearson correlations: Correlations for females above diagonal, correlations for males below diagonal

Variable	SES	TAL	HIP	LIP	SU	Stren PA	Godin (ln)	Days of PA	BMI (ln)
SES		.02	.02	.11	.00	.23*	.24*	.18	-.14
TAL	−.01		.33**	−.36	.66**	−.24*	−.22*	.00	.26*
HIP	.01	.57**		.05	.75**	−.11	−.11	−.04	−.02
LIP	.23*	−.30**	−.04		−.16	.08	.11	−.01	−.31**
SU	.00	.72**	.81**	−.14		−.22*	−.15	−.08	.15
Stren PA	.22	.33**	.17	−.04	.20		.70**	.43**	−.19
Godin (ln)	.26*	.32**	.17	−.12	.19	.83**		.53**	−.12
Days of PA	.21	.24*	.19	−.09	.25*	.48**	.46**		−.11
BMI (ln)	−.01	−.03	.09	.12	.07	−.34**	−.33**	−.05	

*p < .05, **p < .01; SES = Socioeconomic status; TAL = Temperamental activity level; HIP = High intensity pleasure; LIP = Low intensity pleasure; SUR = Surgency; Stren PA = Strenuous physical activity; ln = natural log transformation

**Table 4:** Model results; temperament dimensions added separately to baseline covariate models

	Males				Females				Temp by Sex Interaction
*Days of PA*	*B*	*SE*	*p*	Odds Ratio	*B*	*SE*	*p*	Odds Ratio	
Race^a^	.14	.48	.777	1.02	−.38	.41	.352	0.86	--
SES	**.46**	.24	.054	**1.07**	.27	.19	.165	1.19	--
BMI	−.07	.21	.748	0.87	−.24	.19	.208	0.92	--
TAL	.40	.25	.108	1.30	−.04	.18	.824	0.96	n. s.
HIP	−.08	.21	.699	1.11	−.24	.19	.217	1.01	n. s.
LIP	−.08	.21	.712	0.91	−.27	.20	.177	0.90	n. s.
SUR	***.47***	.23	.043	***1.61***	−.17	.19	.378	1.01	*B* = −.67, *SE* = .30, *p* = .023
***Godin Score***	***B***	***SE***	***p***		***B***	***SE***	***p***		
Race^a^	−.06	.26	.831	--	−.23	.20	.252	--	--
SES	***.27***	.12	.025	--	**.18**	.10	.061	--	--
BMI	***−.37***	.12	.002	--	−.08	.11	.424	--	--
TAL	***.42***	.13	.001	--	−.03	.09	.774	--	*B* = .45, *SE* = .16, *p* = .005
HIP	***.27***	.12	.028	--	−.04	.09	.655	--	*B* = .31, *SE* = .15, *p* = .044
LIP	−.20	.15	.179	--	−.14	.11	.218	--	Main effect significant for combined sample
SUR	***.31***	.12	.010	--	−.05	.10	.617	--	*B* = .34, *SE* = .15, *p* = .024
***Strenuous PA***	***B***	***SE***	***p***	**% Change**	***B***	***SE***	***p***	**% Change**	
Race^a^	.02	.14	.876	2.02	−.15	.16	.328	−13.93	--
SES	.07	.06	.298	7.25	***.17***	.07	.020	***18.53***	--
BMI	**−.14**	.07	.059	**−13.06**	−.08	.09	.373	−7.69	--
TAL	***.26***	.09	.003	***29.69***	−.04	.07	.623	−3.92	*B* = .30, *SE* = .11, *p* = .009
HIP	.10	.07	.177	10.52	.01	.07	.849	1.01	n. s.
LIP	−.09	.08	.262	−8.61	−.11	.08	.177	−10.42	n. s.
SUR	**.14**	.07	.051	**15.03**	.01	.07	.927	1.01	n. s.

^a^0 = Caucasian, 1 = African American and other; bolded values represent *p* < .10; bolded and italicized values represent *p* < .05; PA = Physical activity; TAL = temperamental activity level; HIP = high intensity pleasure; LIP = low intensity pleasure; SUR = surgency; n. s. = non-significant at *p* < .05

YRBS Days of PA. Days of involvement in PA were not significantly predicted by any covariates. The effect of SUR differed significantly by sex such that SUR predicted days of PA in males but not in females. A one standard deviation increase in childhood surgency for males was associated with a 1.61 increase in the odds of being in a higher adolescent PA category (0, 1–4, 5–7 days; CI: 1.02, 2.54). Associations among each of TAL, HIP, and LIP and days of PA did not differ by sex. The proportional odds assumption test did not find evidence that the model suffered misfit due to the use of ordered logistic regressions to model the multiple levels of the dependent variable (all *p* > .05).

Overall PA (Godin Total Score). Male youth from higher SES backgrounds were more likely to be engaged in PA. This effect was only significant at the statistical trend level (*p* = .06) in females. Males with greater BMI were less likely to be engaged in PA. However, BMI was not associated with overall PA in females. Next, we tested predictions from individual childhood dimensions of temperament to overall PA and whether they differed by sex. Males and females differed significantly in their slopes predicting PA from TAL, HIP, and SUR, but not from LIP. Specifically, in males, predictions of overall PA were significant for TAL, HIP, and SUR, but not for LIP. In females, none of these dimensions of temperament were significant predictors of overall PA. Male-only models explained more variance in overall PA than female-only models (Male R^2^ range: .20–.28; Female R^2^ range: .09–.10). When variance accounted for by covariates was subtracted, dimensions of temperament in male-only models explained more variance in overall PA than female-only models (Male ΔR^2^ range: .03–.11; Female ΔR^2^ range: .01–.02).

**Fig. 2:**
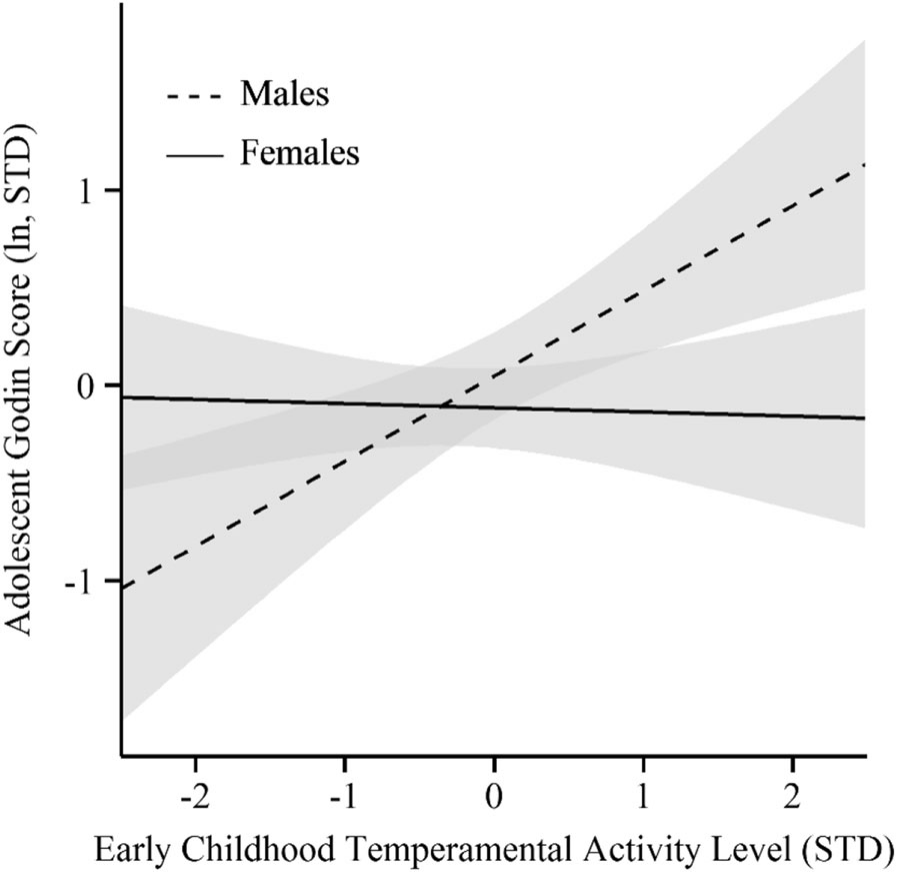
Overall physical activity level as a function of early childhood temperamental activity level; grey bars represent 95% confidence intervals; STD = standardized

Strenuous PA was positively predicted by SES in females but not in males. The prediction from temperament to strenuous PA differed by sex for TAL. No differences in predictions were identified for HIP, LIP, or SUR. In males, a one SD increase in childhood TAL predicted 29.7% more bouts of strenuous PA in adolescence, but no other predictors were significant. In females, childhood temperament did not predict involvement in strenuous activity during adolescence. Figure 2 illustrates the TAL x sex interaction and shows predicted values of strenuous PA as a function of childhood TAL in males and females. The model-implied strenuous PA values did not differ significantly from the observed values. Pearson’s chi-square tests for all models predicting strenuous PA were nonsignificant (*p* > .05) indicating that the models fit the data well.

## Discussion

The current study is among the first longitudinal examinations of associations between childhood temperament and adolescent PA. Broadly, our results suggest that childhood temperamental activity level, high intensity pleasure, and surgency predict adolescent overall PA volume (Godin score) in males. Surgency also predicted the weekly number of days of PA in males. These long-term predictions from childhood temperamental predispositions for activity, intensity preferences, and surgency to PA bouts over a decade later is remarkable, especially given that the transitions of childhood and adolescence are marked by multiple and substantial changes in context. These contextual changes include variations in access to PA opportunities, schools, peers, and family dynamics over time. Our findings suggest that despite these many changes, temperament exerts a lasting influence and may be an underlying driver of PA tracking across the lifespan.

Developmental science and child development theoretical frameworks suggest both direct and indirect pathways through which childhood temperament exerts its long-term influence on PA [e.g., 17, 18, 36]. For example, direct child effects refer to mechanisms through which children actively structure their developmental contexts in ways that fit their individual characteristics. Accordingly, childhood temperament may directly influence children’s choices of activities and friends (e.g., friends with similar interests in physical activity). In addition, indirect child effects may occur when children’s temperamental characteristics influence how others respond to them [e.g., 17]. For example, caregivers of temperamentally active children may observe the child’s need to move and encourage them to participate in extracurricular activities or set aside time for the child to be physically active. These proposed mechanisms cannot be tested in the present study. Broadly however, it is quite possible that dispositions related to temperamental activity level, high intensity pleasure and surgency exert their influence via similar direct and indirect effects on children’s developmental contexts to ultimately contribute to youth’s PA engagement.

Although males’ temperament predicted number of weekly PA bouts and overall PA, early childhood preferences for low intensity pleasure did not negatively predict adolescent PA in either sex. Young children’s preferences for high and low intensity pleasure were uncorrelated, indicating that these predispositions are distinct—which is consistent with previous findings [23]. It is possible that preference for low intensity stimuli may be related to sitting time or other sedentary pursuits, which have been shown to be independently associated with future chronic disease risk [37].

Contrary to our hypothesis, we did not find that strenuous PA was predicted by preference for high intensity pleasure in either boys or girls. We had expected children’s high intensity inclination to influence the preferred intensity of PA that children would perform. Our results may point to differences between the psychological construct of high intensity pleasure—which includes interest in incongruity and novelty of stimuli—and the characterization of physiological intensity as assessed by the Godin score. Strenuous PA questionnaire items provide a measure of activities that are physiologically strenuous such as running. However, these activities are not necessarily psychologically incongruous or stimuli provoking. It is possible that childhood high intensity pleasure may be more likely to facilitate participation in invasion sports (i.e. soccer, basketball), which provide greater amounts of unpredictability, rather than generally physiologically challenging activity. Unexpectedly, we found that childhood temperamental activity level was the sole predictor of male adolescent strenuous activity. Overall, this indicates that engagement in strenuous PA may be driven more by general activity level patterns than by preference for activities of high psychological intensity.

Correlates and predictors of PA are highly influenced by the life stage studied. The lack of prediction from temperament to PA in females in the current study may, in part, be due to the considerable drop in PA levels occurring in females during the adolescent years; a drop that is not paralleled to the same extent in males at this age [38–40]. Cognitive factors (e.g., beliefs) contextual factors (e.g., peers, cultural expectations) and self-confidence may become more important drivers of PA in adolescent females [40]. Indeed, direct and indirect mechanisms for the influence of temperament on PA may differ between sexes. For example, parents may be more inclined to involve their temperamentally active son, rather than their temperamentally active daughter, on a sports team, which, in turn could strengthen the association between temperament and adolescent PA in males, but weaken this association in females.

Our lack of prediction from females’ temperament to later PA was in contrast to previous cross-sectional work that had identified associations between temperamental activity and energy expenditure in a sample of 8-12-year-old females [24]. That study, however, was based on a different population (i.e., older children selected for non-obesity), and also a larger number of females, which makes direct comparisons with results from our study difficult. For example, Anderson and colleagues used energy expenditure measured using doubly labeled water as an outcome variable, whereas the present study uses self-reported bouts of PA. It may be that, in females, temperament influences energy expenditure through increasing PA, and also via non-exercise activity thermogenesis such as fidgeting type behavior, which was not a focus of the present study. Overall, additional work is needed to understand sex differences in the association between temperament and PA at different points in development.

## Strengths and limitations

The present study has several strengths. First, the longitudinal design of over a decade and the interdisciplinary nature of the team provided a unique opportunity to study the prospective association between childhood temperament and later PA. There are few longitudinal samples of this size that track individuals from early childhood into adolescence and with study designs that incorporate both self-regulatory/temperament constructs in childhood and health behaviors such as PA later in life. Second, the CBQ is among the most widely used temperament measures and has received extensive reliability and validation [41]. Parent reports can provide insight into typical child behavior across a wide variety of contexts and eliciting situations that may not be captured by laboratory or time- and context-limited naturalistic observations. Third, the recruitment strategy at age 2 resulted in a participant pool that was highly diverse and representative of the general community.

Despite these strengths, our study is not without limitations. First, due to the novelty of the research question regarding relationships between temperament and PA, our study had a somewhat narrow focus on flushing out these associations, and alternative social and environmental determinants and correlates of PA, including physical and family-based environments, child self-efficacy, parental health behaviors etc., were not measured here. Future work should seek to understand how temperament interacts with additional environmental factors that influence the development of one’s PA behavior. Second, parent reports of child temperament may be biased by limitations of memory, expectations about child behavior, and differences in the reference groups that parents use to report on the relative frequency with which target children engage in specific activities (e.g., child’s siblings, other children in their neighborhood). Third, self-reported measures of PA have lower validity and capture fewer nuances compared to objective assessments of PA [42]. Yet, with little existing literature in the area of temperament and PA, self-report methodology could be considered a sufficient first step. Both the Godin and YRBS have acceptable psychometric properties when compared with existing instruments [29] and results from these measures converged in the present analysis.

Finally, it is possible that associations between childhood temperament and later physical activity are due to genetic mechanisms that were not assessed here. The heritability of temperament is estimated to be between 20–60% [43]; for physical activity, heritability estimates range between 25–60% [44]. Understanding the genetic basis for complex behaviors such as PA and/or temperament is challenging (genetic x environment interactions), but potential overlap between the heritable components of both physical activity and temperament is a plausible explanation for at least parts of the observed associations. It is unclear, however, to what extent this mechanism could explain the sex differences in associations identified in the current study.

Our results point to several additional future directions that could improve understanding of temperamental influences on PA: 1) Study the associations between temperament and PA at earlier ages (including infancy), to provide an understanding of the formation of PA behaviors; 2) Investigate whether there are associations between temperament and PA in females earlier in childhood, prior to the adolescent drop in female PA levels; 3) Observe whether findings are replicated using objective measures of PA such as accelerometry, and if dimensions of temperament predict sedentary bouts, light intensity PA, non-exercise activity thermogenesis, and postural transitions – outcomes that are best assessed with objective techniques; 4) Expand the model of the current study to examine whether temperamental profiles predict chronic disease, and conduct formal tests of mediation to understand whether PA (and other health behaviors) serve as potential mechanisms in this pathway, 5) Examine whether caregiver perspectives of children’s temperamental dispositions influence children’s placement into PA activities and whether these influences differ by sex.

## Conclusion

The focus on temperament provides a unique perspective on individual characteristics that may influence lifelong PA, and may thus have implications for disease risk and prevention. Understanding the key dimensions of temperament that influence PA may help identify high-risk individuals in early childhood for targeted intervention. With current models of temperament suggesting a strong biological component, it may be important to find ways to entice males with low temperamental activity towards engagement in PA pursuits that appeal to their preferred levels of stimulation. Our results suggest that closer examination of early childhood dispositions may provide a promising avenue for understanding the development and maintenance of PA habits into adolescence and, potentially, adulthood.

## Abbreviations

BMI: Body mass index
CBQ: Children’s behavior questionnaire
HIP: High intensity pleasure
LIP: Low intensity pleasure
PA: Physical activity
SES: Socioeconomic status
SUR: Surgency
TAL: Temperamental activity level
YRBS: Youth behavior risk survey
